# Crystal Structure of Human SSRP1 Middle Domain Reveals a Role in DNA Binding

**DOI:** 10.1038/srep18688

**Published:** 2015-12-21

**Authors:** Wenjuan Zhang, Fuxing Zeng, Yiwei Liu, Chen Shao, Sai Li, Hui Lv, Yunyu Shi, Liwen Niu, Maikun Teng, Xu Li

**Affiliations:** 1 Hefei National Laboratory for Physical Sciences at Microscale, Innovation Center for Cell Signaling Network, School of Life Science, 96 Jinzhai Road, Hefei, Anhui, 230026, People’s Republic of China; 2 Key Laboratory of Structural Biology, Hefei Science Center of CAS, Chinese Academy of Sciences, 96 Jinzhai Road, Hefei, Anhui, 230026, People’s Republic of China

## Abstract

SSRP1 is a subunit of the FACT complex, an important histone chaperone required for transcriptional regulation, DNA replication and damage repair. SSRP1 also plays important roles in transcriptional regulation independent of Spt16 and interacts with other proteins. Here, we report the crystal structure of the middle domain of SSRP1. It consists of tandem pleckstrin homology (PH) domains. These domains differ from the typical PH domain in that PH1 domain has an extra conserved βαβ topology. SSRP1 contains the well-characterized DNA-binding HMG-1 domain. Our studies revealed that SSRP1-M can also participate in DNA binding, and that this binding involves one positively charged patch on the surface of the structure. In addition, SSRP1-M did not bind to histones, which was assessed through pull-down assays. This aspect makes the protein different from other related proteins adopting the double PH domain structure. Our studies facilitate the understanding of SSRP1 and provide insights into the molecular mechanisms of interaction with DNA and histones of the FACT complex.

To overcome the inhibitory effects of nucleosomes in the accessibility of DNA during basic cellular processes, such as transcription, DNA replication and repair, many factors are required to alter the chromatin structure. The FACT (facilitates chromatin transcription) complex is an important histone chaperone that reorganizes nucleosomes without hydrolysing ATP^1^. The yeast FACT complex enhances DNA accessibility in nucleosomes without translocating histone octamers relative to the DNA^2^,^3^, whereas the human FACT complex is proposed to function as a histone chaperone that displaces a single H2A-H2B dimer from a nucleosome to promote DNA accessibility^4^,^5^,^6^. The FACT complex is a chromatin-specific factor that is required for both normal regulation of transcription^1^,^7^,^8^,^9^ and DNA replication^9^,^10^,^11^,^12^. It also plays important roles in DNA damage repair^13^,^14^,^15^,^16^. Consistent with its broad functional importance, the FACT complex is a highly conserved complex among all eukaryotes^5^,^11^,^17^,^18^. In *S. cerevisiae*, the FACT complex contains three proteins: ySpt16, Pob3 and Nhp6^1^. In metazoans, the Pob3 and Nhp6 orthologs are fused to form a single polypeptide called SSRP1. Both *SPT16* and *SSRP1/Pob3* are essential genes for the viability of eukaryotes^9^,^19^. hSpt16 binds to H2A-H2B dimers and nucleosomes. In contrast, SSRP1 binds neither H2A-H2B dimers nor nucleosomes, but it does bind H3-H4 tetramers^4^.

The domain structure of the FACT complex is conserved throughout eukaryotes. Spt16 is composed of four domains, including an N-terminal domain (Spt16-N), a dimerization domain (Spt16-D), a middle domain (Spt16-M) and a C-terminal domain (Spt16-C)^20^,^21^. SSRP1 contains three well-defined domains: an N-terminal/dimerization domain (SSRP1-N), a middle domain (SSRP1-M) and a C-terminal high-mobility group (HMG)-1 domain (SSRP1-C)^21^. Pob3 includes two domains similar to SSRP1-N and SSRP1-M with a C-terminal acidic region. Nhp6 contains an HMG-l domain. Recently, the structures and the functions of the domains of the FACT complex were revealed^12^,^18^,^20^,^22^,^23^,^24^. Briefly, it was found that ySpt16-N, which is structurally similar to aminopeptidases, binds to histones H3-H4^18^. ySpt16-M and Pob3-M both adopt tandem PH domain topologies^20^,^22^,^23^. ySpt16-M binds to H2A-H2B and H3-H4 histones^22^,^23^ whereas Pob3-M binds to only H3-H4 histones^22^,^25^. Notably, a related yeast histone H3-H4 chaperone Rtt106, which has sequence homology with Pob3 and SSRP1, has been identified^26^, and it contains a double PH domain^25^,^27^,^28^. Nhp6A adopts a typical L-shaped HMG fold and binds nonspecifically and stably to dsDNA^24^. However, no structural studies on the human FACT complex have been reported, and its ability to bind DNA and histone remains to be elucidated.

SSRP1 was initially identified as binding to specific DNA modified by the anticancer drug cisplatin^29^. The protein is expressed at high levels in proliferating cancerous tissues and at low levels in less-renewable and differentiated tissues^30^,^31^. Furthermore, SSRP1 can bind to cruciform DNA, linear duplex DNA and cisplatin-modified DNA^32^,^33^,^34^. SSRP1 has also been reported to act as an architectural transcription factor for many other genes^33^. Additionally, SSRP1 can function as a co-activator of several transcription factors, such as serum-response factor (SRF)^35^ and p63^36^. In addition, SSRP1 is a novel microtubule (MT)-binding protein that facilitates MT growth and bundling, and it is required for mitosis^37^. However, the mechanisms by which SSRP1 binds to DNA and interacts with other proteins and how each domain of SSRP1 contributes to the functions of SSRP1 are unclear. Among the three domains of SSRP1, the ability of the C-terminal HMG domain of SSRP1 to bind DNA has been studied most intensively^16^,^34^,^38^. However, the other domains of SSRP1 (SSRP1- (N + M)) are even more greathighly conserved than the HMG domain^32^,^39^, suggesting their importance for the functions of SSRP1. Consistently, SSRP1- (N + M) domains have been found to be critical for the interaction between SSRP1 and chromatin^40^. Although the N-terminal domain of SSRP1, (SSRP1-N), was reported to be required for the *in vitro* interaction of SSRP1 with Spt16^41^, little is known about the middle domain of SSRP1 (SSRP1-M), which is highly conserved across species (Fig. 1). In the present work, we determined the crystal structure of SSRP1-M at a resolution of 1.93 Å. The structure contains tandem pleckstrin homology (PH) domains and exhibits a positively charged region on each side. Furthermore, we investigated the dsDNA binding properties of this domain and tested its interaction with histones via biochemical assays.

## Materials and Methods

### Protein expression and purification

cDNA fragments encoding SSRP1-N (residues 1–176), SSRP1-M (residues 196–430), and SSRP1-C (residues 513–709) were PCR-amplified from the full-length SSRP1 cDNA (Sanying). Each of the cDNAs was cloned into a modified pET-28a (+) plasmid lacking a thrombin cleavage site to produce target proteins as N-terminal 6 × His fusions. cDNA encoding the gene for Rtt106 was also cloned into the modified pET-28a (+) plasmid as reported^27^ for *in vitro* His-tag pull-down assays. cDNA encoding SSRP1-M or HIF was cloned into the pGEX-4T-2 vector to produce the GST-fused protein for *in vitro* GST pull-down assays. Mutations were generated using a QuikChange Site-directed Mutagenesis kit (Stratagene) according to the manufacturer’s protocol. All of the recombinant plasmids were confirmed by DNA sequencing (Invitrogen) and then transformed into *Escherichia coli* BL21 (DE3) (Novagen), for protein expression. *E. coli* cells were cultured in LB medium with 50 mg/l kanamycin or 100 mg/l ampicillin at 310 K until the OD600 reached 0.8–1.2. Then, the cells were induced by the addition of 0.25 mM isopropyl-1-thio-β-D-galactopyranoside (IPTG) at 289 K for 20 h. The cells were then collected by centrifugation, resuspended in buffer A (20 mM Tris-HCl, 200 mM NaCl, 5 % glycerol, pH 7.0) and lysed via ultrasonication. The cell extracts were centrifuged at 14,000 × g for 30 min at 277 K. The supernatants were purified using Ni^2+^-nitrilotriacetate or GST affinity resin (GE Healthcare) pre-equilibrated with buffer A. The eluted proteins were concentrated via centrifugal ultrafiltration (Amicon Ultra-15, 10 kDa MWCO, Millipore). The concentrated poor was loaded onto a pre-equilibrated HiLoad 16/60 Superdex 200 column or a HiLoadTM 16/60 SuperdexTM 75 pg column (GE Healthcare) using an Äkta-System (GE Healthcare), and the protein was eluted at a flow rate of 1 ml/min with buffer B (20 mM Tris-HCl, 200 mM NaCl, pH 7.0) and collected in 1.2 ml fractions. The peak fractions were analysed using Tricine-SDS-PAGE (15 %, w/v) and stained with Coomassie brilliant (CB) blue R250. The purified fractions were pooled together and concentrated via centrifugal ultrafiltration. For crystallization, SSRP1-M was further purified by HiTrap SPFF (5 ml) ion exchange chromatography (GE Healthcare). The final protein pool in buffer C (5 mM Tris-HCl, 40 mM NaCl, pH 7.5) was concentrated to 40 mg/ml for crystallization trials.

### Crystallisation and data collection

SSRP1-M was crystallized using the hanging drop vapour diffusion method by mixing together 1 μl protein solution and 1 μl reservoir solution at 287 K. The crystal with suitable X-ray diffraction was grown in a reservoir solution that contained 0.1 M Tris, and 15 % (w/v) polyethylene glycol 6,000, pH 8.5 (Hampton Research). Data collection was performed at 100 K with a cryoprotectant solution containing the reservoir solution with the addition of 20 % (v/v) glycerol. A set of diffraction data was collected using the beamline BL17U of the Shanghai Synchrotron Radiation Facility (SSRF).

### Structure determination and refinement

The diffraction data set was processed and scaled using the *HKL2000* package^42^. The phases were determined using molecular replacement with the program *Molrep*^43^. The structure of Pob3-M (PDB code 2GCL) was used as the search model. Cycles of refinement and model building were carried out using the *REFMAC5*^44^, *Phenix*^45^ and *COOT*^46^ software programs until the crystallography R-factor and free R-factor converged to 19.8 % and 21.7 %, respectively. The Ramachandran analysis showed that 92.7 % of the residues were in the most favoured region, and that an additional 7.3 % were within the allowed region. The quality of the structure was verified using the program *MolProbity*^47^. The details regarding the data collection and processing are summarized in Table 1. All the structural figures were prepared using *PyMOL* (DeLano Scientific).

### Electrophoretic mobility shift assay (EMSA)

The protein-DNA interactions were evaluated using an EMSA. The DNA sequences used are as follows:

30-bp linker dsDNA, 5′-TCCAGTGCCGGTGTCGCTTGGGTCCCGAGG-3′;

AT-rich dsDNA, 5′-ATAATTTATATTTATTATTTTATTATAATT-3′;

GC-rich dsDNA, 5′-GCGGCCCGCGCCCGCCGCCCCGCCGCGGCC-3′;

The DNA segments were synthesized by Sangon (Shanghai). Two strands of complementary single-stranded DNA were annealed to form the dsDNA. Next, 8 μM dsDNA was mixed with increasing amounts of protein from 10–320 μM in buffer D (20 mM Tris-HCl, 200 mM NaCl, 1 mM DTT, 1 mM EDTA, pH 7.0) and then incubated on ice for 1 h to be resolved on a 1.0 % agarose gel at 60 V and 4 °C in 1 × TAE buffer (40 mM Tris-acetate, 1 mM EDTA, pH 7.8), or an 8 % native polyacrylamide gel at 100 V and 4 °C in 0.5 × TBE buffer (45 mM Tris-borate, 1 mM EDTA, pH 8.0). The gel was visualized using Gel-Red. The amount of DNA was estimated using ImageJ software^48^. The raw integrated intensity was plotted against the protein concentration.

### *In vitro* pull-down assays

The plasmids encoding the Xenopus histones H2A, H2B, H3 and H4 were kind gifts from Dr. Guohong Li^49^. The fragments encoding the yeast histones H2A, H2B, H3 and H4 were amplified from yeast genomic cDNAs using PCR, and the plasmids were made as described previously^50^. The histones were bacterially expressed, and the histone octamers were reconstituted using an established protocol^51^. Whole calf thymus histones were purchased from Worthington Company. The *in vitro* pull-down assays were performed as described previously^52^. Briefly, 10 μg of GST or GST-fused SSRP1-M or GST-fused HIF was bound to 20 μl of glutathione-Sepharose beads (GE Healthcare). Then, the beads were incubated with reconstituted histone octamers or whole calf thymus histones in a binding buffer containing 50 mM Tris-HCl, 1 M NaCl, and 0.2 % Triton X-100 at pH 7.5 for 30 min at room temperature. The protein-bound glutathione resins were washed five times with the binding buffer, and the bound samples were subjected to SDS-PAGE. The gels were stained using Coomassie Blue. His-tag pull-down assays were carried out similarly, with the exceptions that His-tagged SSRP1 or His-tagged Rtt106 was used on Ni^2+^-nitrilotriacetate resins and reconstituted yeast histone octamers were input for Rtt106.

## Results

### Crystal structure of the middle domain of SSRP1

**Figure 1:**
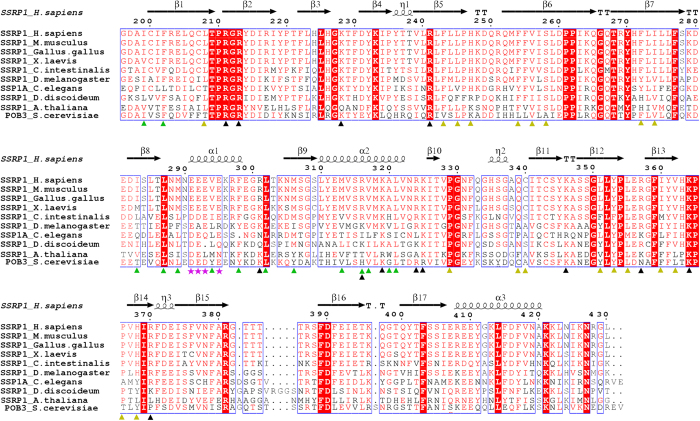
Sequence alignment of SSRP1-M with its homologues. The amino acid sequences of the 10 indicated species from human to yeast are aligned, with the secondary structural elements reported in this paper indicated above the sequences. The amino acids involved in the hydrophobic inter-domain interactions are designated with yellow triangles; residues involved in stabilizing the extra βαβ super-secondary structure are designated with green triangles; residues comprising the positively charged regions for dsDNA binding are designated with black triangles; and residues comprising the unique negatively charged regions are designated with magentas stars.

**Figure 2:**
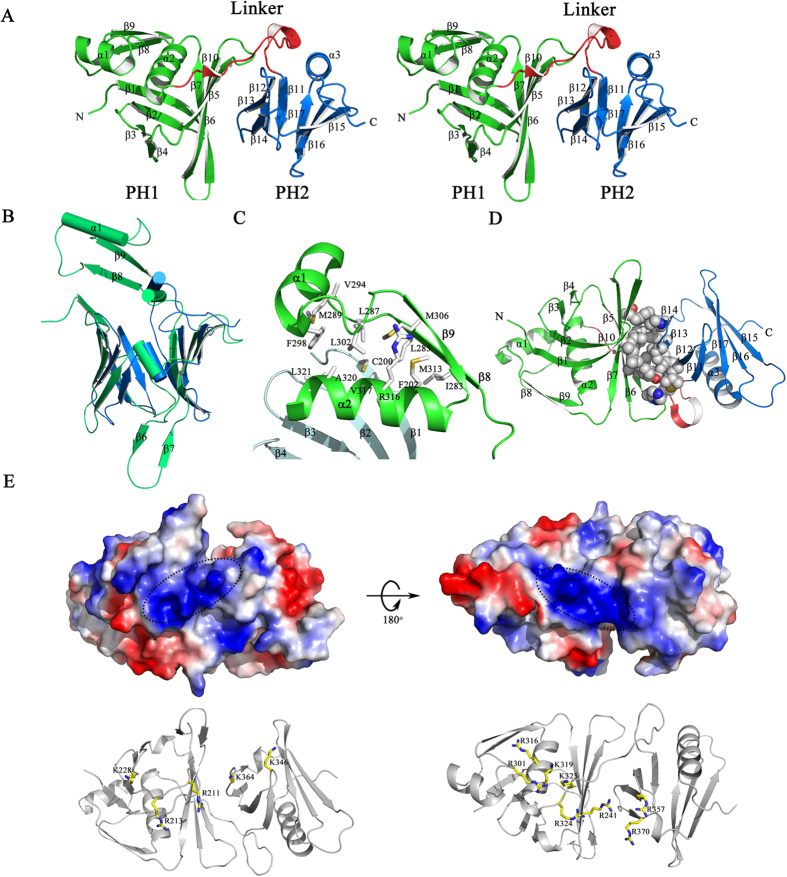
Structural features of SSRP1-M. (**A**) A stereoscopic representation of SSRP1-M. The secondary structural elements are labelled. The structure is coloured according to the domains, as follows: the PH1 domain (residues 197–323), green; the inter-domain linker (residues 324–340), red; and the PH2 domain (residues 341–427), blue. (**B**) The structural superposition of PH1 domain (green) and PH2 domain (blue) of SSRP1-M with the differences labelled. (**C**) The extra βαβ topology of the PH1 domain stabilized by hydrophobic interactions. (**D**) The hydrophobic contacts between the PH1 domain and PH2 domain. The involved residues are indicated as spheres coloured by atom-type (O, red; N, blue; C and H, grey). (**E**) Upper panel, the electronic potential surface of SSRP1-M. The two positively charged patches are highlighted. Lower panel, the basic residues comprising the positively charged patches.

The crystal structure of the middle domain of SSRP1 (SSRP1-M) was determined at a resolution of 1.9 Å using molecular replacement; one molecular in the asymmetric unit is consistent with the observation that SSRP1-M is eluted as a monomer during the size exclusion chromatography of the purification process. The crystallographic statistics are summarized in Table 1. The structure contains tandem pleckstrin homology (PH) domains (Fig. 2A). In particular, the second PH domain (PH2) highly resembles the typical PH domain, which comprises of a seven-stranded antiparallel β-barrel capped by a C-terminal helix^53^. Superposition of the SSRP1-M PH1 domain (residues 197–328) with the PH2 domain (residues 329–427) revealed a root-mean-square (r.m.s.) deviation of 2.4 Å on 89 pairs of Cα atoms (Fig. 2B). In comparison with the second PH domain, the PH1 domain contains two extra antiparallel strands (β8 and β9) linked by a helix (α1), which are inserted between the last strand of the PH domain (β7) and the β-barrel-capping helix (α2). This, leads to a better-sealing cap. In addition, in the other corner of the β-barrel, the much longer β-strands (β6 and β7) in the PH1 domain form a tighter interaction composed of three inter-strand loops, which are usually potential binding sites. The extra secondary structure is stabilized by its hydrophobic interactions with residues that reside on the β1 and α2 of PH1, as shown in the enlarged view in Fig. 2C. The hydrophobic residues, which include I283, L285 and M306 on β8 and β9; M289, V294 and F298 on α1; L287 and L302 on the in-between linker; and M313, R316, V317, A320 and L321 on α2; and C200 and F202 on β1, form an extensive hydrophobic core. Notably, these residues are highly conserved among the homologues of SSRP1 (Fig. 1), suggesting that this extra topology is evolutionarily conserved and may be functionally significant. The two PH domains are associated with each other rather than independent, burying a solvent-accessible surface area of approximately 680 Å^2^. They interact primarily through the hydrophobic contacts mediated by the residues located on β5 to β7 of the PH1 domain and on β2 to β4 of the PH2 domain (Fig. 2D). Interestingly, these residues are also well conserved across species (Fig. 1), suggesting that the compact topology is evolutionarily conserved. The analysis of the electrostatic potential surface of the SSRP1-M structure revealed that there is a positively charged region stretching across the PH domains on each side of SSRP1-M (Fig. 2E, upper panel). Several positively charged residues, including K228, R213, R211, K364 and K346 on one side and R316, R301, K319, K325, R324, R241, R357, and R370 on the other side, constitute the positively charged regions (Fig. 2E, lower panel). Most of these residues are highly conserved (Fig. 1) implying functional relevance.

### SSRP1-M binds to DNA non-specifically

Because arginine and lysine residues are commonly involved in interactions with DNA^24^,^27^, the positively charged regions suggest the DNA-binding activity of SSRP1. It has been reported that SSRP1 binds to linker DNA among nucleosomes^54^, and it is generally attributed to the C-terminal HMG-1 domain, which enables the proteins to bind to DNA as it enters and exits a nucleosome^55^.

**Figure 3:**
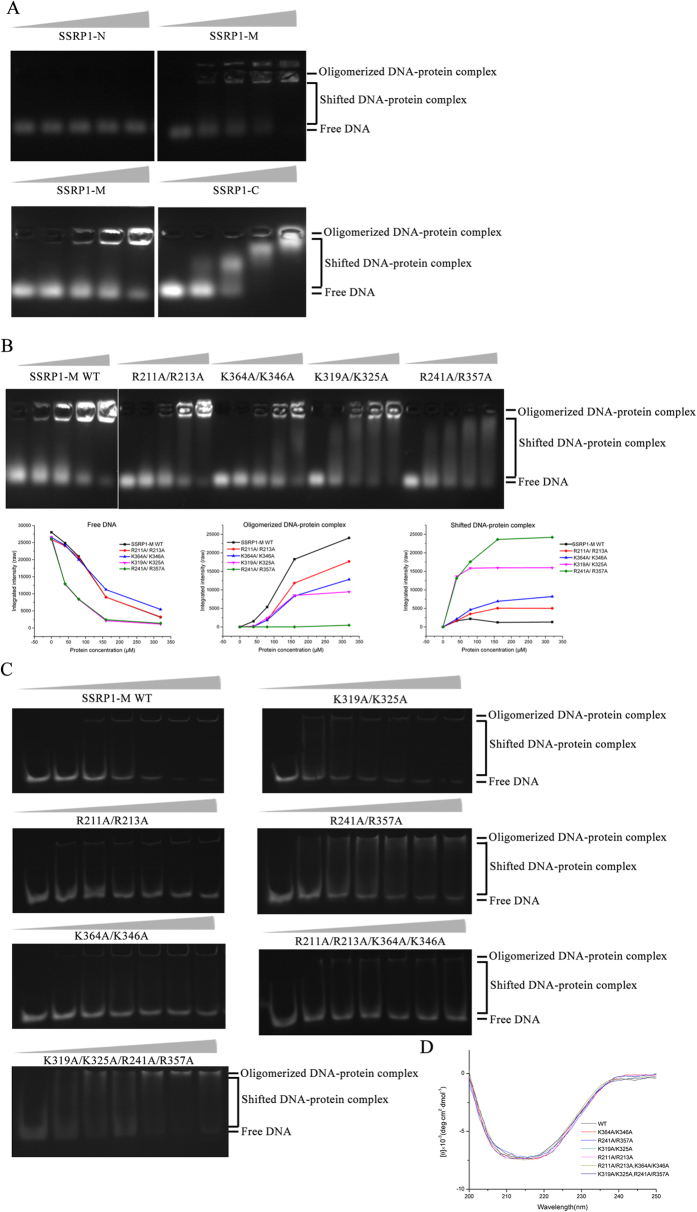
SSRP1-M is able to bind to DNA. (**A**) Agarose EMSA results of different constructs of SSRP1 with 30-bp linker dsDNA. In each lane, 8μM dsDNA was incubated with increasing amounts of protein (0, 40, 80, 160, and 320 μM for SSRP1-N and SSRP1-M, and 0, 10, 20, 40 and 80 μM for SSRP1-C). Upper panel, the gels were stained for 10mins; lower panel, the gels were stained for 15mins. The relevant bands were labelled. (**B**) Upper panel: agarose EMSA results of SSRP1-M and its four mutants, R211A/R213A, K364A/K346A, K319A/K325A and R241A/R357A. The concentration of dsDNA in each lane was 8 μM. The concentrations of protein were the same as SSRP1-M in (**A**). Lower panel: raw quantified curves for the amount of free DNA, oligomerized DNA-protein complex and shifted DNA-protein complex as labeled in each gel against the protein concentration. (**C**) Native polyacrylamide EMSA results of SSRP1-M and its mutants, R211A/R213A, K364A/K346A, K319A/K325A, R241A/R357A, R211A/R213A/K364A/K346A and K319A/K325A/R241A/R357A, with 30-bp linker dsDNA. The concentration of dsDNA in each lane was 8 μM. The concentrations of proteins in each gel were 0, 10, 20, 40, 80, 160, and 320 μM, respectively. (**D**) CD spectra of SSRP1-M and its six mutants as indicated.

To investigate whether SSRP1-M contributes to the DNA binding of SSRP1, we tested the DNA-binding abilities of different constructs of SSRP1 via an agarose gel electrophoretic mobility shift assay (EMSA) using a 30-bp linker dsDNA, which has an identical sequence to the linker regions of the 207-bp nucleosomal DNA (601 sequence)^56^. As shown in the upper panel of Fig. 3A, no obvious change in the amount of free DNA in the gel was observed after the addition of SSRP1-N. For the addition of SSRP1-C, which contains the HMG domain and has been established as a DNA-binder, a shift from the free to the bound form in the gel was observed (Fig. 3A lower panel). Furthermore, the addition of increasing amounts of SSRP1-M caused the amount of free DNA in the gel to progressively decrease, and an increasing amount of DNA oligomerized in the wells (Fig. 3A upper and lower panels). As shown in our figures, the free DNA bands were labelled as “free DNA”; the shifting bands in the gel were labelled as “shifted DNA-protein complex”; and the bands in the well were labelled as “oligomerized DNA-protein complex”.

To investigate why DNA is oligomerized in the well, we mutated some basic residues on each positively charged surface of SSRP1-M to alanine according to its structure and observed their behaviors in gel shift assays. In agarose EMSA (Fig. 3B), all the four mutants caused less oligomerized DNA-protein complex but more shifted DNA-protein complex than the wild-type protein, as judged from both gel graphs and raw quantified curves. While the amount of free DNA at the end of the run is similar for the wild type and R211A/R213A across the protein concentrations tested, the graphs and curves show that K364A/K346A had more free DNA than the wild-type, which indicates that this mutant has reduced DNA-binding ability. K319A/K325 and especially R241A/ R357A, caused the amounts of free DNA and oligomerized DNA-protein complex greatly reduced and correspondingly, the amount of shifted DNA highly increased by comparing with the wild-type protein. The result shows that the two mutants relieved the highly oligomerized DNA-protein complexes by shifting them into smaller complexes, which are able to run in the gel. This finding suggests that the four residues, R319, K325, especially R241 and R357, play key roles in the oligomerization of DNA-protein complexes. For the native polyacrylamide EMSAs (Fig. 3C), the mutants R211A/R213A and K364A/K346A caused more free DNA than the wild-type protein. While a little gel shifting appeared in some lanes with the K319A/K325A mutant, much more shifted DNA-complex was observed in each lane with the mutant R241A/R357A than the wild-type protein. Our results showed that SSRP1-M is able to bind to DNA and form DNA-protein complexes. After binding DNA, these complexes oligomerize to form high molecular oligomers that are retarded in the well region of the gel. Residues R211, R213, especially K364 and K346 are important for the interaction between SSRP1-M and DNA to form a complex, whereas the residues K319, K325, especially R241 and R357, contribute to the high oligomerization of DNA-protein complexes. Consistently, the results of the native polyacrylamide EMSA assay of the four-site mutants showed that the R211A/R213A/K364A/K346A mutant obviously decreased the DNA binding affinity of SSRP1-M, while the R241A/R357A/K319A/K325A mutant caused increasing gel shifting compared with the wild-type protein (Fig. 3C). All of the mutants exhibited similar CD spectra to that of wild-type SSRP1-M (WT), indicating that the mutation did not affect the folding of the proteins (Fig. 3D).

**Figure 4:**
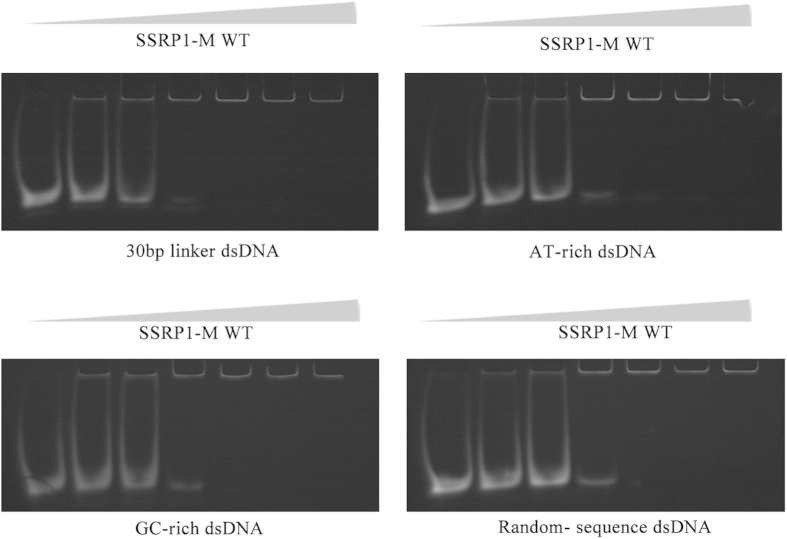
SSRP1-M binds to DNA without a sequence preference. EMSA results of SSRP1-M with four different types of dsDNA, including 30-bp linker dsDNA, an AT- rich sequence, a GC-rich sequence and a random-sequence DNA. In each lane, 8 μM dsDNA was incubated with increasing amounts of SSRP1-M (0, 20, 40, 80, 160, and 320 μM, respectively).

To investigate whether SSRP1-M has a DNA sequence preference, we performed binding studies of SSRP1-M with a 30-bp linker dsDNA, AT- or GC-rich sequences or a random-sequence DNA using EMSA assays. As shown in Fig. 4, no obvious differences in the DNA-binding affinity of SSRP1-M were observed for the different types of dsDNA, indicating that SSRP1-M binds to DNA with no sequence preference.

In summary, our results indicated that SSRP1-M is able to bind to DNA nonspecifically and the positively charged patch, including residues R211, R213, K364 and K346, is important for the DNA-binding ability of SSRP1-M, while the other positively charged patch on the opposite side takes part in the oligomerization of DNA-protein complexes. This is the first time that the DNA-binding ability of SSRP1 has been demonstrated outside the SSRP1-C domain.

### SSRP1-M was unable to bind to histones

**Figure 5:**
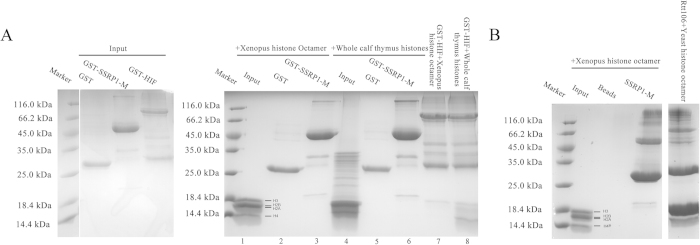
SSRP1-M cannot bind to histones, as determined by *in vitro* pull-down assays. (**A**) The input proteins alone are shown on the left. The results of the GST pull-down assays of SSRP1-M with reconstituted Xenopus histone octamers and whole calf thymus histones are shown on the right, using GST-HIF as a positive control. The bands of the histones are indicated. (**B**) The His-tag pull-down assays of SSRP1-M with reconstituted Xenopus histone octamers, using His-Rtt106 bound to yeast histone octamers as a positive control. The bands of the histones are indicated.

The FACT complex, which is composed of Spt16 and SSRP1, possesses intrinsic histone chaperone activity^4^, and different components of the yFACT complex, including Spt16-N, Spt16-M and Pob3-M, have been reported to bind to histones^18^,^22^,^23^,^25^. However, no study has reported about the histone-binding ability of the individual domains of SSRP1. Therefore, it is of interest to determine whether SSRP1-M binds to histones. First, we performed GST pull-down assays using the recombinant GST fusion proteins. We reconstituted Xenopus histone octamers from recombinant histones and tested the interaction between SSRP1-M and the octamers. By comparison with the proteins on their own, it was found that SSRP1-M does not bind to histones, whereas the HIF protein, which has been reported to selectively interact with H3 and H4 histones^57^, binds to the H3 histone (Fig. 5A, lanes 1–3 and 7). Furthermore, we performed pull-down assays with whole calf thymus histones, and similarly, there was no obvious binding detected for SSRP1-M, in contrast to the HIF protein (Fig. 5A, lanes 4–6 and 8). In addition, we performed His-tag pull-down assays, and the same results were observed. There was no obvious binding detected between SSRP1-M and the *Xenopus* histone octamers. In contrast, Rtt106, which is reported to bind to histone H3-H4^27^, substantially bound to yeast histone octamers (Fig. 5B) under the same experimental conditions. In summary, our pull-down assay results showed that SSRP1-M binds weakly, if at all, to histones compared with HIF and Rtt106.

## Discussion

**Figure 6:**
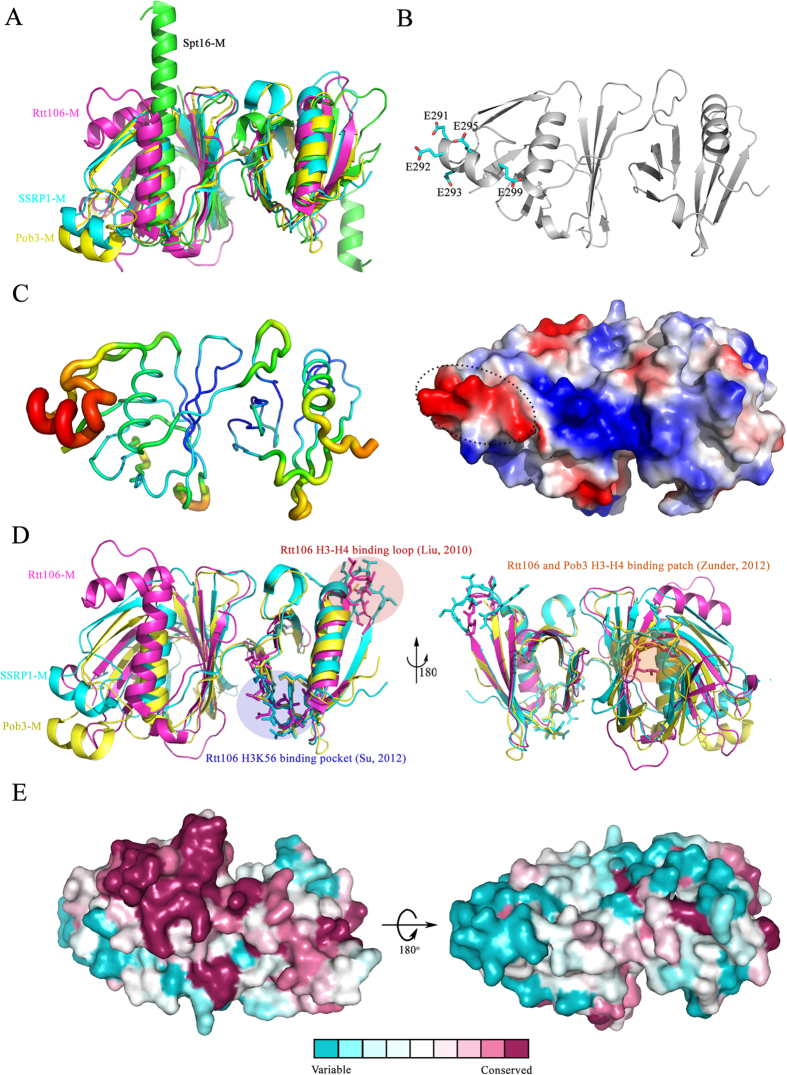
A structural comparison of SSRP1-M with other proteins adopting double PH domains and surface conservation analysis of SSRP1-M. (**A**) The structural superposition of the tandem PH domains of SSRP1-M (cyan, PDB ID: 4IFS), Pob3-M (yellow, PDB ID: 2GCL; average r.m.s.d. of 1.52 Å on 225 Cα-atoms), Rtt106-M (magentas, PDB ID: 3GYP; average r.m.s.d. of 2.44 Å on 174 Cα-atoms), and Spt16-M (green, PDB ID: 4IOY; average r.m.s.d. of 2.55 Å on 178 Cα-atoms). (**B**) Upper panel: the acidic residues comprising the negatively charged region; Lower panel: the electronic potential surface of SSRP1-M with the unique negatively charged region labelled. (**C**) The B-factor distribution of SSRP1-M. The wider and redder tubing indicates higher B-factor. (**D**) The structural superposition of SSRP1-M (PDB ID: 4IFS), Pob3-M (PDB ID: 2GCL) and Rtt106-M (PDB ID: 3GYP). The reported histone binding surfaces of Pob3 and Rtt106 are indicated. (**E**) The surface conservation analysis of SSRP1-M. The orientations are kept the same as Fig. 2E.

Our studies revealed that the middle domain of SSRP1 adopts a compacted structure with tandem PH domain, similar to that of its yeast homologue Pob3 and the related proteins Rtt106-M^27^ and Spt16-M^22^,^23^. By superimposing the structures of these proteins with that of SSRP1-M, we found that the PH2 domains are quite similar, with the exception of an additional C-terminal α-helix in Spt16-M, and that the primary differences within the PH1 domain reside in the region between β7 and the last α-helix (Fig. 6A). Rtt106-M contains an inserted α-helix. In Spt16-M, there is no insertion, but the last α-helix is extended, forming a much longer helix. However, SSRP1-M and Pob3-M contain two extra antiparallel strands linked by a helix. As mentioned above, this extra topology is evolutionarily conserved and may be functionally significant (Fig. 1). In particular, five acidic residues located in the linking helix (E291, E292, E293, E295 and E299) make up a negatively charged region on the electrostatic potential surface of SSRP1-M (Fig. 6B). Most of these residues are highly conserved (Fig. 1), suggesting that they are associated with various biological roles, such as the participation in the reported interaction between SSRP1-M and p63γ^36^ and the interaction among SSRP1-M and α-, β-, and γ-tubulins^37^. Consistently, the linking helix displays a higher temperature factor within the structure, implying flexibility in its function (Fig. 6C). In addition, the structure of the FACT heterodimerization domain was reported to possess a single PH domain in Spt16-D and a tandem PH domain in Pob3-N^22^. The purpose of the duplication of the PH domain within the FACT complex remains to be addressed.

All three proteins, Spt16-M, Pob3-M and Rtt106-M, have been reported to readily bind to the H3-H4 histones^22^,^25^,^28^. Although no structures of the complexes formed by these proteins with the H3-H4 histones have been reported, various regions of the proteins have been identified as important for histone binding. These regions include the basic patch in the PH1 domains of Rtt106-M and Pob3-M^25^, and a loop, and a hydrophobic area within the PH2 domain of Rtt106-M^25^,^27^,^28^. Spt16-M lacks the basic patch in its PH1 domain, but a prominent acidic patch in PH2 may account for the binding^23^. SSRP1-M has the highest sequence identity (38%) with Pob3-M, and the structure of SSRP1-M highly resembles that of Pob3-M, with an r.m.s. deviation of 1.52 Å on the 225 Cα-atoms (Fig. 6D). Similar to Pob3-M, SSRP1-M contains all the identified histone-binding regions of Rtt106-M, except the loop. The corresponding sequences of the loop are similar in SSRP1-M and Pob3-M (the amino acids between β15 and β16 in Fig. 1), although a loop was not observed in the structure of Pob3. However, SSRP-M showed little ability to bind histones in our pull-down assays, in contrast to all the three other proteins. It suggests that although the structures and sequences among SSRP1-M, Pob3-M and Rtt106 are quite similar, the histone binding ability is different. It may stem from the slight difference in human histones and yeast histones leading to the different binding models. Just as shown in Fig. 6D, even for the same protein Rtt106, the histone-binding sites can be different. SSRP1-M may lack the required site not shown here to bind human histones. By comparing the domain structures of SSRP1 and Pob3 plus Nhp6, SSRP1 has one unique intrinsically disordered (IDD) domain just on the C terminal of SSRP1-M^21^,^58^. Considering the flexibility of IDD domain, it may either bind histone alone or assist SSRP1-M to bind histone. The mechanisms of histone binding remain unclear. Therefore, further assays are required to uncover the detailed mechanisms.

The SSRP1 protein is one of many HMG domain-containing proteins, which usually either contain multiple HMG domains or a single HMG domain without any other DNA-binding region^59^,^60^. However, our study showed that SSRP1-M also binds to dsDNA non-specifically. One of the positively charged patches on the surface of SSRP1-M, the R211A/R213A side, participates in the binding of dsDNA binding. The opposite positively charged patch, which includes residues K319, K325, R241 and R357, participates in the high oligomerization of the DNA-protein complexes. Considering that SSRP1-M exists as a monomer as determined by gel filtration, the oligomerization of the DNA-protein complexes is promoted by the addition of DNA. The binding of DNA through the R211A/R213A side may cause the confirmation change of SSRP1-M, which makes the opposite side more exposed to the solvent, inducing the oligomerization among DNA-protein complexes. However, the oligomerization should be only observed for our construct, which makes the R241A/R357A side of SSRP1-M exposed to the solvent. For the *in vivo* full-length SSRP1 protein, such a positively charged patch may bind to other parts of SSRP1 to form a compact protein, avoiding the oligomerization. Furthermore, there is an acidic residues-rich region between the SSRP1-M and SSRP1-C domains^61^, which may interact with the R241A/R357A side of SSRP1-M. When comparing the electronic potential surfaces of Rtt106-M and SSRP1-M, the positively charged patch on the R211A/R213A side of SSRP1-M corresponds to the only positively charged patch on the surface of Rtt106, which plays important roles in the binding of DNA and is essential for its functions^27^. Consistently, the positively charged patch on R211A/R213A side of SSRP1-M is much more conserved than the opposite side according to the surface conservation analysis (Fig. 6E). This suggests that such a positively charged patch on the structure of SSRP1-M might be crucial for the binding of DNA *in vivo* In addition, Pob3-M has also been found to bind to dsDNA^27^, suggesting its conserved role in DNA binding. It has been reported that SSRP1 requires linker DNA to interact stably with the nucleosome and that the presence of linker DNA positively influences the occupancy of the FACT complex^54^. According to our studies, SSRP1-M also directly contributes to the binding of linker DNA, except for the high-affinity DNA binding domain HMG-1. Therefore, SSRP1-M may act as an important fragment that helps SSRP1 and the FACT complex bind to DNA or chromatin. It was reported that during apoptosis, SSRP1-C is released from chromatin, while SSRP1- (N + M) remains bound to Spt16 in a tight association with chromatin^40^. The nonspecific DNA-binding ability of SSRP1-M may contribute to the binding of the truncated FACT complex to chromatin at this stage. Given the multi-domains of SSRP1, the domains may interact with each other, thus intermolecularly masking the C-terminal HMG-1 domain. When Spt16 is present to bind to SSRP1-N^13^,^21^, a conformational change occurs that unveils the HMG domain of SSRP1, which is consistent with the report that the presence of Spt16 notably increases the nucleosomal DNA binding of SSRP1 and even the FACT complex^16^,^54^. The DNA-binding ability of SSRP1-M may also contribute to other DNA-binding functions of SSRP1 independent of Spt16, such as transcriptional regulation^32^.

In summary, in this study, we present the crystal structure of SSRP1-M. We uncovered its dsDNA-binding ability, which is in addition to the well-recognized ability of the HMG-1 domain to bind DNA, for the first time. Although SSRP1 is known to bind H3-H4 histones^4^, we found that the SSRP1-M domain does not substantially bind to histones. Our structural and biochemical studies of SSRP1-M shed light on the functions of the multiple domains in the SSRP1 protein and facilitate the understanding of the molecular mechanisms of interaction with DNA and histones of the FACT complex.

## Additional Information

**Accession codes:** Atomic coordinates and structure factors for human SSRP1 middle domain have been deposited in the Protein Data Bank under the accession codes 4IFS.

**How to cite this article**: Zhang, W. *et al.* Crystal Structure of Human SSRP1 Middle Domain Reveals a Role in DNA Binding. *Sci. Rep.* **5**, 18688; doi: 10.1038/srep18688 (2015).

## Figures and Tables

**Table 1:** Data-Collection and Refinement Statistics for SSRP1-M.

Data collection	
Space group	*P*3_2_21
Cell dimensions	*a *= *b* = 89.19 Å, *c* = 82.59 Å
Wavelength (Å)	0.97954 Å
Resolution (Å)	50.0 − 1.93 (1.96 − 1.93)^a^
R_merge_ (%)^c^	8.7 (49.6)^a^
I/σ (I)	26.6 (4.9)^a^
CC (1/2)	0.994 (0.970)
Completeness (%)	99.9 (100)^a^
Redundancy	10.7 (10.8)
**Refinement**	
Resolution (Å)	36.4 − 1.93
Number of reflections/test	27578/1476
R_work_/R_free_ (%)^c^	19.8/21.7
Number of atoms Protein	1883
Number of waters	97
Rmsd bond (Å)	0.009
Rmsd angle (°)	1.116
B-value (Å^2^)	39.8
**Ramachandran plot (%)**	
most favoured	92.7
additional allowed	7.3

^a^Values in parentheses are for the highest-resolution shell.

^b^R_merge_ = ∑|I_i_ − <I>|/∑|I|, where I_i_ is the intensity of an individual reflection and <I> is the average intensity of that reflection.

^c^R_work_ = ∑||F_o_| − |F_c_||/∑|F_o_|, where F_o_ and F_c_ are the observed and calculated structure factors for reflections, respectively.

^d^R_free_ was calculated as R_work_ using the 5% of reflections that were selected randomly and omitted from refinement.
